# Online carbohydrate 3D structure validation with the *Privateer* web app

**DOI:** 10.1107/S2053230X24000359

**Published:** 2024-01-24

**Authors:** Jordan S. Dialpuri, Haroldas Bagdonas, Lucy C. Schofield, Phuong Thao Pham, Lou Holland, Paul S. Bond, Filomeno Sánchez Rodríguez, Stuart J. McNicholas, Jon Agirre

**Affiliations:** a York Structural Biology Laboratory, Department of Chemistry, University of York, York YO10 3BG, United Kingdom; Centro Nacional de Biotecnología – CSIC, Spain

**Keywords:** *Privateer*, validation, polysaccharides, carbohydrates, *N*-glycosylation, *N*-glycans, web apps

## Abstract

Owing to the difficulties associated with working with carbohydrates, validating glycan 3D structures prior to deposition into the Protein Data Bank has become a staple of the structure-solution pipeline. The *Privateer* software provides integrative methods for the validation, analysis, refinement and graphical representation of 3D atomic structures of glycans, both as ligands and as protein modifiers. While *Privateer* is free software, it requires users to install any of the structural biology software suites that support it or to build it from source code. Here, the *Privateer* web app is presented, which is always up to date and available to be used online (https://privateer.york.ac.uk) without installation. This self-updating tool, which runs locally on the user’s machine, will allow structural biologists to simply and quickly analyse carbohydrate ligands and protein glycosylation from a web browser whilst retaining all confidential information on their devices.

## Abstract 2

The *Privateer* carbohydrate 3D structure-validation software is now freely available as a web app. Here, its use is described, including a practical example.

## 1. Introduction

Modelling protein glycosylation is an often-overlooked aspect in the field of structural biology. The underappreciation of oligosaccharide modelling can partially be explained by the challenges encountered during the model-building stage. These challenges are primarily caused by the very nature of the carbohydrates: branched, flexible and prone to register microheterogeneity. This results in a tangible impact on protein structures: the median resolution for glycoproteins (2.4 Å) is lower than that of nonglycosylated proteins (2.0 Å) when X-ray crystallography electron-density maps are considered (van Beusekom *et al.*, 2018 ▸). As a result, many glycoprotein models deposited in the wwPDB contain flaws ranging from minor irregularities to gross modelling errors (Agirre, Davies *et al.*, 2015 ▸; van Beusekom *et al.*, 2018 ▸; Crispin *et al.*, 2007 ▸; Lütteke *et al.*, 2004 ▸; Bagdonas *et al.*, 2020 ▸). These difficulties extend to ligand polysaccharide structures as well, with additional complications due to the non-negligible likelihood of finding both α and β anomeric ring forms (Agirre, 2017 ▸), and potentially even linear forms, at the reducing end.

To counteract these issues, carbohydrate-validation software packages have been developed and are currently actively being developed to automate the detection and rectification of modelling errors. Some notable examples are legacy tools such as *pdb-care* (PDB CArbohydrate REsidue check) that are used for glycosidic bond and nomenclature validation; the tool utilizes the *pdb*2*linucs* algorithm, which generates LINUCS notations for modelled oligosaccharides in the model file, which are later compared with HET records in the PDB file (Lütteke *et al.*, 2004 ▸). *CARP* (CArbohydrate Ramachandran Plot) is a tool that can be used to evaluate glycosidic linkage torsions and compare them against data deposited in GlyTorsionDB or GlycoMapsDB (Lütteke *et al.*, 2005 ▸). These tools, which are no longer available, were originally developed and made available as web servers and, while this made them easy to access and run, it detached them from widely used structure-solution software suites such as *Phenix* (Liebschner *et al.*, 2019 ▸) and *CCP*4 (Agirre *et al.*, 2023 ▸). Being remote services, they also relied on data uploads, prompting confidentiality concerns for many industrial users.

The *Privateer* software (Agirre, Iglesias-Fernández *et al.*, 2015 ▸) was originally introduced to detect and address issues affecting the ring conformation of pyranosides (Agirre, Davies *et al.*, 2015 ▸) using a combination of metrics such as Cremer–Pople puckering parameters (Cremer & Pople, 1975 ▸), the real-space correlation coefficient (Atanasova *et al.*, 2022 ▸), average *B* factors and nomenclature checks. It was subsequently extended to implement features of the aforementioned legacy tools in a modern setting (Agirre, Iglesias-Fernández *et al.*, 2015 ▸; Agirre, 2017 ▸). *Privateer* is now capable of performing the automated validation of carbohydrate-ring conformation, monosaccharide nomenclature, glycosidic linkage stereochemistry and torsional conformation (Dialpuri *et al.*, 2023 ▸). Another core aspect of *Privateer* is the capability to compute omit *mF*_o_ − *DF*_c_ maps that are used to calculate a correlation coefficient between carbohydrate residues and input experimental density. *Privateer* can also be used in the context of structure refinement, as it is able to generate chemical dictionaries with unimodal torsion restraints which aid in keeping pyranosides in their lowest energy conformations during refinement; this feature has been used to aid in the low-resolution refinement of large glycoprotein structures (Gristick *et al.*, 2017 ▸) and prompted the creation of updated dictionaries for pyranose sugars (Atanasova *et al.*, 2022 ▸). Finally, *Privateer* can be used to qualitatively assess the compositional properties of modelled oligosaccharides in the input model file by querying the glycan composition in the GlyTouCan repository and the GlyConnect database (Bagdonas *et al.*, 2020 ▸). The most relevant validation information is visually summarized in vector diagrams that are compliant with the Symbol Nomenclature for Glycans (SNFG), where potential modelling errors are highlighted to the user (Haltiwanger, 2016 ▸).

The core functionality of *Privateer* is written in the C++ language, and all of the functionality that allows the user to validate atomic models can be accessed by simply running its executable, or binary (the privateer command). One of the downsides of running the binary is that as a user it can be difficult or even impossible to harness many of the analytical capabilities of *Privateer*. To compensate for this, *Privateer* also has a rich set of Python bindings that allow other programs to utilize the validation tools contained within its shared library (libprivateer.so). Both of these methods require a user to download and compile the source code, or use a software suite such as *CCP*4 (Agirre *et al.*, 2023 ▸) or *CCP-EM* (Burnley *et al.*, 2017 ▸), which not every glycobiologist will have available.

One of the most accessible methods of distributing code is on the web, where users can freely interact with any functionality without the steep learning curve associated with downloading and compiling code (to access the most up-to-date version of the software) or using a software suite. Here, we present a new way to use *Privateer* through a completely client-side web interface running within the user’s web browser. By using *Privateer* online, structural biologists can efficiently validate carbohydrates on any modern operating system or device: desktop and laptop computers, and mobile devices.

## 2. Materials and methods

The source code of *Privateer* uses C++ as the main language for speed-critical operations and Python for higher level functions and scripting; Python functions and types stem from C++ functions wrapped using PyBind11. The primary repository in which this source is kept is hosted at GitHub (https://github.com/glycojones/privateer); due to the marked differences between a typical binary distribution of *Privateer* and the web app presented here, a decision was made to create a separate branch (‘webapp’) for the web app. Crucially, the *Privateer* web app is automatically compiled, built, packaged and deployed on the web server (https://privateer.york.ac.uk) using GitHub Actions, which are triggered the moment new features are ready to be released. Therefore, the *Privateer* web app is always up to date with the latest developments and fixes.

The connection between the *Privateer* source code in C++ and the web interface is made possible using the compilation tool *Emscripten*. This tool is able to compile C++ code into WebAssembly, an assembly language that is compatible with most modern web browsers. Using *Emscripten*, parts of *Privateer* are compiled into WebAssembly and are made accessible using a set of JavaScript bindings in a method that is similar to that of the Python bindings already present in *Privateer*. In addition to this, to allow for more functionality, the dependencies of *Privateer* must also be compiled into WebAssembly libraries and bundled in.

The interface of the website is created using *React* (https://react.dev/), which allows a flexible and dynamically loadable site to be built and deployed statically. *React* was chosen to enhance compatibility with the 3D visualization software used in this project, *Moorhen* (https://moorhen.org). *Moorhen* is web-based molecular-graphics software based on the *Coot* desktop application (Emsley *et al.*, 2010 ▸); *Moorhen* and *Coot* rely on the functionality encapsulated in the *Coot* libraries, and therefore are expected to produce similar results. Like the *Privateer* web app, *Moorhen* is compiled into WebAssembly and runs locally on the user’s browser. The *Moorhen* panel displayed on the report page presents only a relevant subset of the *Moorhen* interface, including controls for changing the map level, showing symmetry mates and activating Glycoblocks (McNicholas & Agirre, 2017 ▸), a 3D extension of the Standard Symbol Nomenclature for Glycans, commonly known as SNFG (Neelamegham *et al.*, 2019 ▸). A brief overview of the controls can be found by pressing ‘h’ on the *Moorhen* panel. Finally, while the *Moorhen* panel has all of the functionality of the full web application, users are invited to run a standalone *Moorhen* session to work on models comfortably. This may be performed easily either by accessing the *Moorhen* website (https://moorhen.org) or by running it from *CCP*4 *Cloud* (Krissinel *et al.*, 2022 ▸).

## 3. Results and discussion

**Figure 1:**
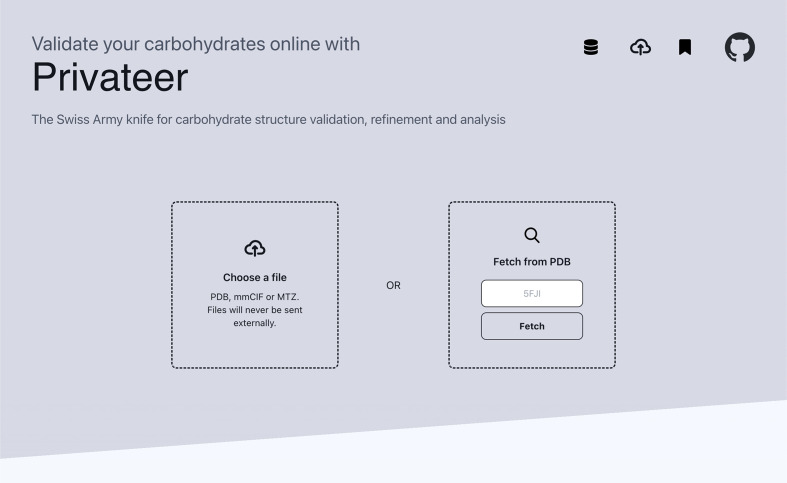
Input screen of the *Privateer* web app. The user is asked to either supply a structure or a structure code, which is then verified against the Protein Data Bank (wwPDB Consortium, 2019 ▸) and downloaded from it.

The *Privateer* web app has two main data-entry points: the user either chooses a local file or specifies a PDB code to be fetched from the Protein Data Bank (wwPDB Consortium, 2019 ▸), as shown in Fig. 1 ▸ (also please refer to our Supplementary Video, which demonstrates the case study presented here). This allows the web app to function as both a validation tool during structure determination and a resource for the analysis of deposited structures. When using the web app as a validation tool, a user can choose a single coordinate file in PDB or mmCIF format for geometric validation and, in addition, an accompanying reflection file for further density-fitness analysis as measured by RSCC, the real-space correlation coefficient (Atanasova *et al.*, 2022 ▸). Once the correct files have been selected, *Privateer* is used to evaluate known carbohydrates found in the coordinate file.

**Figure 2:**
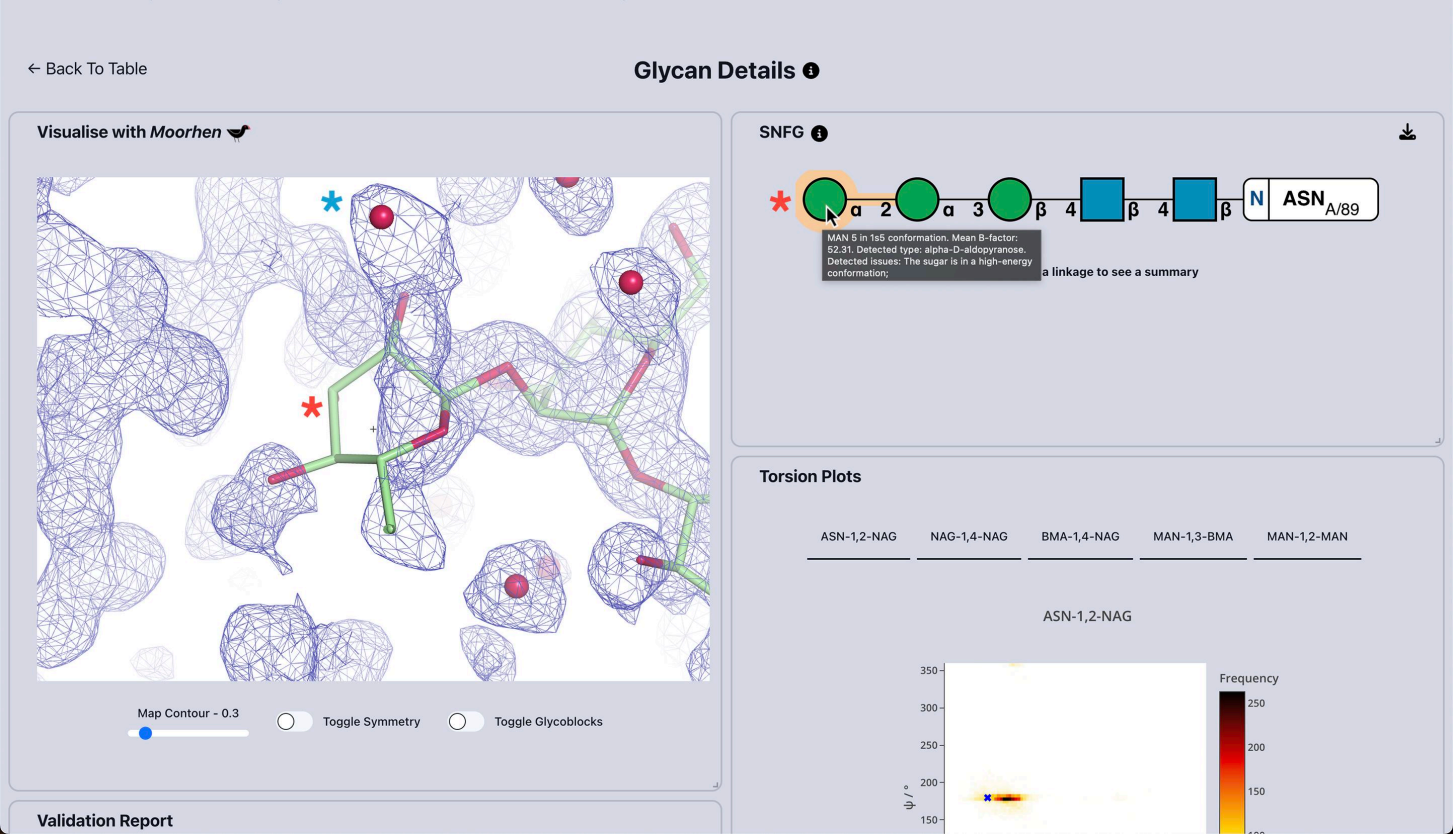
Detailed view of the glycan chain linked to Asn89 and the *Moorhen* visualization panel. The terminal mannose (red asterisk) can be seen to be in a ^1^ *S*_5_ high-energy conformation as opposed to the expected ^4^ *C*_1_ conformation. Additionally, it has multiple atoms outside the 2*mF*_o_ − *F*_c_ density, presumably due to a clash with the adjacent water molecule (blue asterisk), which lies at the other end of the electron density for the mannose.

The web app displays its results in a table with the different glycans. Each table entry contains information for every carbohydrate found in the provided structure, including the chain ID, GlyTouCan (Fujita *et al.*, 2021 ▸) identifier and a diagram in the Standard Symbol Nomenclature For Glycans (Haltiwanger, 2016 ▸). This information and the SNFG diagrams allow the rapid identification of a particular oligosaccharide group. Clicking on an entry displays more information about the glycan, as shown in Fig. 2 ▸.

## 4. Data security

Perhaps one of the most common reasons not to use the available web servers for carbohydrate structure validation is the potential lack of data security. Confidential structures are sent to a third-party server to be validated, which is likely to be forbidden by many industrial organizations. With a client-side web app such as that presented here, user data are never sent externally; in fact, once the site content is completely loaded, an internet connection is no longer required. By default, however, the site is loaded dynamically to prevent slow initial page loads.

## 5. A case study: validating, correcting and extending a structure

We will use the high-resolution (1.2 Å) crystal structure of glucose oxidase (PDB entry 3qvp; Kommoju *et al.*, 2011 ▸) as an example. This was originally modelled with a five-sugar *N*-glycan chain linked to Asn89, as well as three single *N*-acetyl­glucosamine pyranosides linked to Asn161, Asn355 and Asn388. The electron-density map is very clear, as expected for such high resolution, and the global quality indicators show that the overall quality of the model is excellent.

The glycan summary of PDB entry 3qvp generated using the ‘Fetch from PDB’ input box on the *Privateer* web app shows a table with the detected glycans. From this table view, it is simple to identify any glycans that contain any modelling anomaly by looking for orange highlights around a linkage or sugar icon. In the case of PDB entry 3qvp, the single GlcNAc glycans are deemed to be modelled within the expected parameters, whereas the α1,2-linked mannose sugar and glycosidic linkage require further inspection. Clicking on the first table entry reveals more information about this glycan and its potential issues (Fig. 2 ▸). The validation data for this glycan attached to Asn89 via an N atom highlights a single conformational issue with the α1,2-linked mannose (red asterisk in Fig. 2 ▸). Due to a clash with an adjacent water molecule (blue asterisk in Fig. 2 ▸) that lies in the continuous electron density of the mannose, this sugar has been distorted into a ^1^ *S*_5_ conformation as opposed to the expected ^4^ *C*_1_ chair conformation. Most likely as a result of this high-energy ring conformation, the α1,2 link (also highlighted in orange) has uncommon torsion angles.

The *Privateer* web app displays these conformational and torsional issues within the SNFG as pop-up messages when the mouse hovers over a sugar or a linkage; however, it is more commonplace for a structural biologist to want to visualize the glycan using desktop model-visualization software. The inbuilt *Moorhen* visualization panel removes the need to open locally installed visualization software and allows trivial inspection of the glycan chain: users need only to click on the sugars for the 3D graphics window to re-centre on them. Using this visualization, it is clear that the α1,2-linked mannose sugar is indeed in a high-energy conformation and is additionally not modelled within the density. This monosaccharide is a strong candidate for remodelling and subsequent refinement.

To resolve this conformational anomaly, the mannose and neighbouring modelled water molecules were deleted in *Coot* (Emsley *et al.*, 2010 ▸). An α1,2 link was added to a new ^4^ *C*_1_ mannose, which was then refined with *REFMAC*5 (Murshudov *et al.*, 2011 ▸). Following refinement, the density of two further α-mannose sugars could be identified. One was attached to the rebuilt α1,2-linked mannose, and another was attached to the β1–4 mannose, which were then modelled and refined.

**Figure 3:**
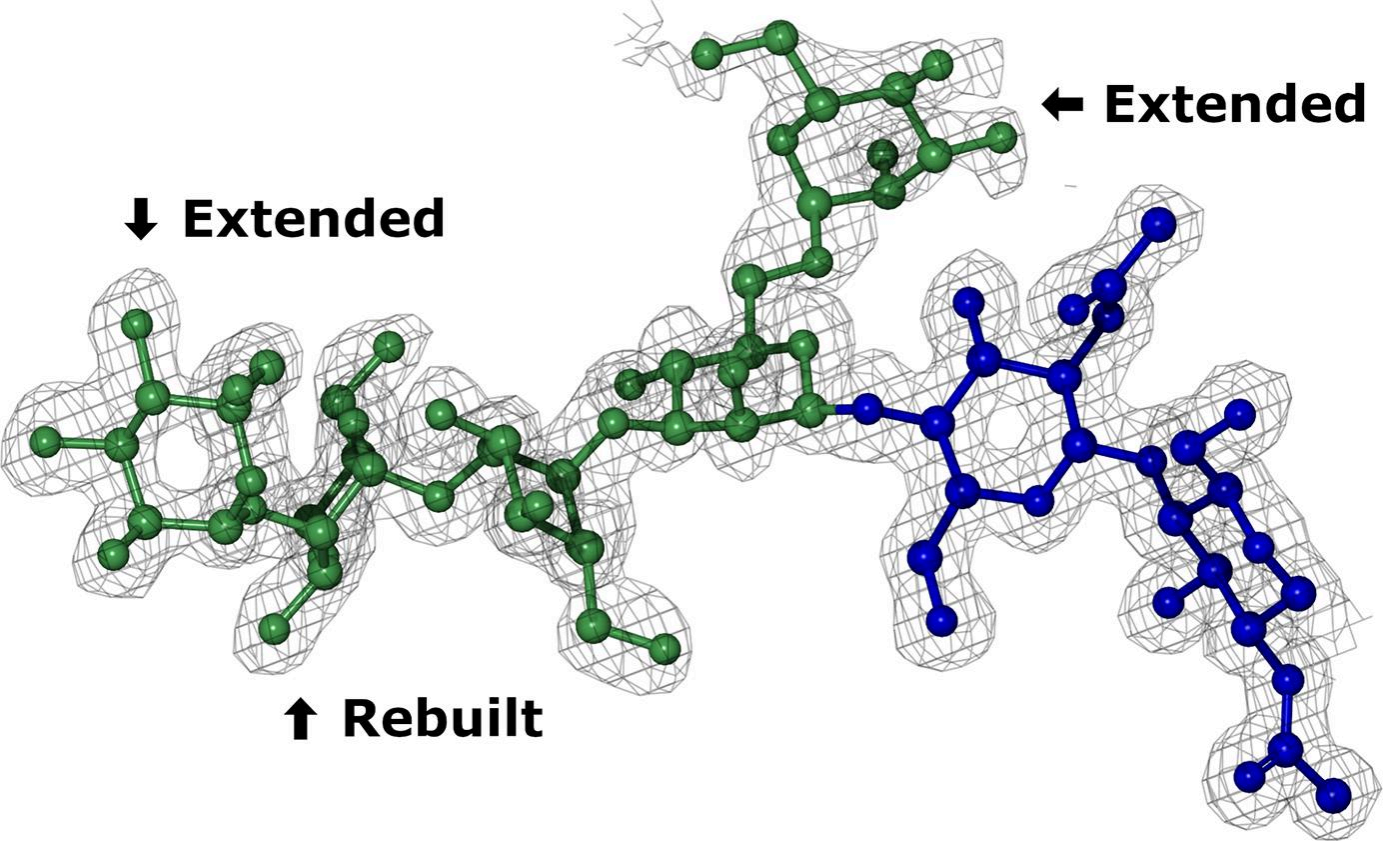
The refined structure of the rebuilt *N*-glycan. 2*mF*_o_ − *F*_c_ density is contoured at 1σ. The individual monosaccharides have been orientated and coloured according to the updated SNFG diagram shown in Fig. 4 ▸ (left panel). The annotations show the rebuilt and extended monosaccharides. This figure was generated with *CCP*4*mg* (McNicholas *et al.*, 2011 ▸).

**Figure 4:**
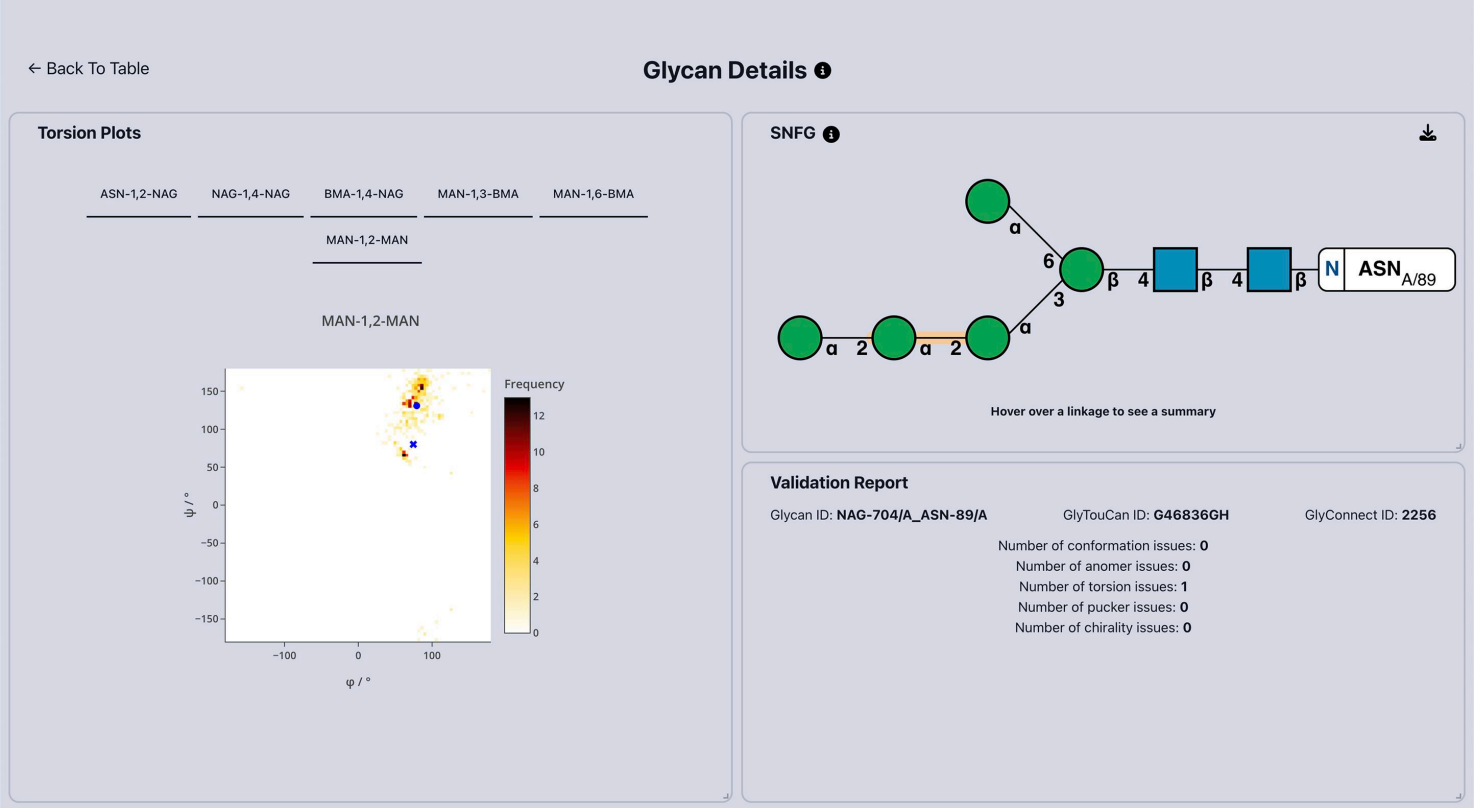
Right: detailed information about the glycan attached to Asn89 in PDB entry 3qvp (Kommoju *et al.*, 2011 ▸) after remodelling and refinement. Two additional α-mannose sugars were added to the chain and the conformational issues of the deposited α1,2-mannose were corrected. Left: linkage torsion angles panel, which highlights the current torsion angles for this linkage (blue crosses) in relation to the previously reported torsional landscape.

This updated structure (Fig. 3 ▸) was finally analysed using the web app, with only the original torsional issue remaining. Further inspection of this issue can be performed using the torsion plots that are also available in the detailed glycan view page (Fig. 4 ▸). Upon inspection of the MAN-1,2-MAN torsion tab, the highlighted linkage is very close to the expected clusters and hence is little cause for concern. This assertion is validated by inspection of the linkage in the *Moorhen* visual­ization panel, which shows a good density fit.

## 6. Conclusions

In conclusion, the *Privateer* web app is an innovative online tool for carbohydrate 3D structure validation. The web app allows fast, local structure validation without the requirement to send any files to an external service. Harnessing the functionality of *Privateer*, users can validate structural composition and conformation, anomericity and linkage-torsion outliers from a web browser.

## 7. Availability and reproducibility

A video demonstrating the *Privateer* web app is available as supporting information. All source code is publicly available on GitHub at https://github.com/glycojones/privateer. The original, updated structure and map coefficients in MTZ format are available as supporting information. The *Privateer* web app is available at https://privateer.york.ac.uk and will remain automatically updated with respect to the source code on GitHub.

## Supplementary Material

Rebuilt and refined structure (PDB entry 3qvp). DOI: 10.1107/S2053230X24000359/va5056sup1.zip

Video demo of the Privateer web app. DOI: 10.1107/S2053230X24000359/va5056sup2.mp4

## References

[bb1] Agirre, J. (2017). *Acta Cryst.* D**73**, 171–186. 10.1107/S2059798316016910 PMC5297920 28177313

[bb2] Agirre, J., Atanasova, M., Bagdonas, H., Ballard, C. B., Baslé, A., Beilsten-Edmands, J., Borges, R. J., Brown, D. G., Burgos-Mármol, J. J., Berrisford, J. M., Bond, P. S., Caballero, I., Catapano, L., Chojnowski, G., Cook, A. G., Cowtan, K. D., Croll, T. I., Debreczeni, J. É., Devenish, N. E., Dodson, E. J., Drevon, T. R., Emsley, P., Evans, G., Evans, P. R., Fando, M., Foadi, J., Fuentes-Montero, L., Garman, E. F., Gerstel, M., Gildea, R. J., Hatti, K., Hekkelman, M. L., Heuser, P., Hoh, S. W., Hough, M. A., Jenkins, H. T., Jiménez, E., Joosten, R. P., Keegan, R. M., Keep, N., Krissinel, E. B., Kolenko, P., Kovalevskiy, O., Lamzin, V. S., Lawson, D. M., Lebedev, A. A., Leslie, A. G. W., Lohkamp, B., Long, F., Malý, M., McCoy, A. J., McNicholas, S. J., Medina, A., Millán, C., Murray, J. W., Murshudov, G. N., Nicholls, R. A., Noble, M. E. M., Oeffner, R., Pannu, N. S., Parkhurst, J. M., Pearce, N., Pereira, J., Perrakis, A., Powell, H. R., Read, R. J., Rigden, D. J., Rochira, W., Sammito, M., Sánchez Rodríguez, F., Sheldrick, G. M., Shelley, K. L., Simkovic, F., Simpkin, A. J., Skubak, P., Sobolev, E., Steiner, R. A., Stevenson, K., Tews, I., Thomas, J. M. H., Thorn, A., Valls, J. T., Uski, V., Usón, I., Vagin, A., Velankar, S., Vollmar, M., Walden, H., Waterman, D., Wilson, K. S., Winn, M. D., Winter, G., Wojdyr, M. & Yamashita, K. (2023). *Acta Cryst.* D**79**, 449–461.

[bb3] Agirre, J., Davies, G., Wilson, K. & Cowtan, K. (2015). *Nat. Chem. Biol.* **11**, 303. 10.1038/nchembio.1798 25885951

[bb4] Agirre, J., Iglesias-Fernández, J., Rovira, C., Davies, G. J., Wilson, K. S. & Cowtan, K. D. (2015). *Nat. Struct. Mol. Biol.* **22**, 833–834. 10.1038/nsmb.3115 26581513

[bb5] Atanasova, M., Nicholls, R. A., Joosten, R. P. & Agirre, J. (2022). *Acta Cryst.* D**78**, 455–465. 10.1107/S2059798322001103 PMC8972801 35362468

[bb6] Bagdonas, H., Ungar, D. & Agirre, J. (2020). *Beilstein J. Org. Chem.* **16**, 2523–2533. 10.3762/bjoc.16.204 PMC7554661 33093930

[bb7] Beusekom, B. van, Lütteke, T. & Joosten, R. P. (2018). *Acta Cryst.* F**74**, 463–472. 10.1107/S2053230X18004016 PMC6096482 30084395

[bb8] Burnley, T., Palmer, C. M. & Winn, M. (2017). *Acta Cryst.* D**73**, 469–477. 10.1107/S2059798317007859 PMC5458488 28580908

[bb9] Cremer, D. & Pople, J. A. (1975). *J. Am. Chem. Soc.* **97**, 1354–1358.

[bb10] Crispin, M., Stuart, D. I. & Jones, E. Y. (2007). *Nat. Struct. Mol. Biol.* **14**, 354. 10.1038/nsmb0507-354a 17473875

[bb11] Dialpuri, J. S., Bagdonas, H., Atanasova, M., Schofield, L. C., Hekkelman, M. L., Joosten, R. P. & Agirre, J. (2023). *Acta Cryst.* D**79**, 462–472. 10.1107/S2059798323003510 PMC10233620 37219590

[bb12] Emsley, P., Lohkamp, B., Scott, W. G. & Cowtan, K. (2010). *Acta Cryst.* D**66**, 486–501. 10.1107/S0907444910007493 PMC2852313 20383002

[bb13] Fujita, A., Aoki, N. P., Shinmachi, D., Matsubara, M., Tsuchiya, S., Shiota, M., Ono, T., Yamada, I. & Aoki-Kinoshita, K. F. (2021). *Nucleic Acids Res.* **49**, D1529–D1533. 10.1093/nar/gkaa947 PMC7779025 33125071

[bb14] Gristick, H. B., Wang, H. & Bjorkman, P. J. (2017). *Acta Cryst.* D**73**, 822–828. 10.1107/S2059798317013353 PMC5633907 28994411

[bb15] Haltiwanger, R. S. (2016). *Glycobiology*, **26**, 217. 10.1093/glycob/cww034 27122386

[bb16] Kommoju, P., Chen, Z., Bruckner, R. C., Mathews, F. S. & Jorns, M. S. (2011). *Biochemistry*, **50**, 5521–5534. 10.1021/bi200388g PMC3448946 21568312

[bb17] Krissinel, E., Lebedev, A. A., Uski, V., Ballard, C. B., Keegan, R. M., Kovalevskiy, O., Nicholls, R. A., Pannu, N. S., Skubák, P., Berrisford, J., Fando, M., Lohkamp, B., Wojdyr, M., Simpkin, A. J., Thomas, J. M. H., Oliver, C., Vonrhein, C., Chojnowski, G., Basle, A., Purkiss, A., Isupov, M. N., McNicholas, S., Lowe, E., Triviño, J., Cowtan, K., Agirre, J., Rigden, D. J., Uson, I., Lamzin, V., Tews, I., Bricogne, G., Leslie, A. G. W. & Brown, D. G. (2022). *Acta Cryst.* D**78**, 1079–1089. 10.1107/S2059798322007987 PMC9435598 36048148

[bb18] Liebschner, D., Afonine, P. V., Baker, M. L., Bunkóczi, G., Chen, V. B., Croll, T. I., Hintze, B., Hung, L.-W., Jain, S., McCoy, A. J., Moriarty, N. W., Oeffner, R. D., Poon, B. K., Prisant, M. G., Read, R. J., Richardson, J. S., Richardson, D. C., Sammito, M. D., Sobolev, O. V., Stockwell, D. H., Terwilliger, T. C., Urzhumtsev, A. G., Videau, L. L., Williams, C. J. & Adams, P. D. (2019). *Acta Cryst.* D**75**, 861–877. 10.1107/S2059798319011471 PMC6778852 31588918

[bb19] Lütteke, T., Frank, M. & von der Lieth, C.-W. (2004). *Carbohydr. Res.* **339**, 1015–1020. 10.1016/j.carres.2003.09.038 15010309

[bb20] Lütteke, T., Frank, M. & von der Lieth, C.-W. (2005). *Nucleic Acids Res.* **33**, D242–D246. 10.1093/nar/gki013 PMC539967 15608187

[bb21] McNicholas, S. & Agirre, J. (2017). *Acta Cryst.* D**73**, 187–194. 10.1107/S2059798316013553 PMC5297921 28177314

[bb22] McNicholas, S., Potterton, E., Wilson, K. S. & Noble, M. E. M. (2011). *Acta Cryst.* D**67**, 386–394. 10.1107/S0907444911007281 PMC3069754 21460457

[bb23] Murshudov, G. N., Skubák, P., Lebedev, A. A., Pannu, N. S., Steiner, R. A., Nicholls, R. A., Winn, M. D., Long, F. & Vagin, A. A. (2011). *Acta Cryst.* D**67**, 355–367. 10.1107/S0907444911001314 PMC3069751 21460454

[bb24] Neelamegham, S., Aoki-Kinoshita, K., Bolton, E., Frank, M., Lisacek, F., Lütteke, T., O’Boyle, N., Packer, N. H., Stanley, P., Toukach, P., Varki, A., Woods, R. J., Darvill, A., Dell, A., Henrissat, B., Bertozzi, C., Hart, G., Narimatsu, H., Freeze, H., Yamada, I., Paulson, J., Prestegard, J., Marth, J., Vliegenthart, J. F. G., Etzler, M., Aebi, M., Kanehisa, M., Taniguchi, N., Edwards, N., Rudd, P., Seeberger, P., Mazumder, R., Ranzinger, R., Cummings, R., Schnaar, R., Perez, S., Kornfeld, S., Kinoshita, T., York, W. & Knirel, Y. (2019). *Glycobiology*, **29**, 620–624. 10.1093/glycob/cwz045 PMC7335484 31184695

[bb25] wwPDB Consortium (2019). *Nucleic Acids Res.* **47**, D520–D528. 10.1093/nar/gky949 PMC6324056 30357364

